# Bifurcation Asymmetry of Small Coronary Arteries in Juvenile and Adult Mice

**DOI:** 10.3389/fphys.2018.00519

**Published:** 2018-05-15

**Authors:** Yundi Feng, Xuan Wang, Tingting Fan, Li Li, Xiaotong Sun, Wenxi Zhang, Minglu Cao, Jian Liu, Jianping Li, Yunlong Huo

**Affiliations:** ^1^Department of Mechanics and Engineering Science, College of Engineering, Peking University, Beijing, China; ^2^Department of Cardiology, Peking University People's Hospital, Beijing, China; ^3^Department of Cardiology, Peking University First Hospital, Beijing, China

**Keywords:** coronary arterial tree, bifurcation asymmetry, bifurcation angle, advancing age, mouse model

## Abstract

**Background:** Microvascular bifurcation asymmetry is of significance for regulation of coronary flow heterogeneity during juvenile and adult growth. The aim of the study is to investigate the morphometric and hemodynamic variation of coronary arterial bifurcations in mice of different ages.

**Methods:** Pulsatile blood flows were computed from a Womersley-type model in the reconstructed left coronary arterial (LCA) trees from Micro-CT images in normal mice at ages of 3 weeks, 6 weeks, 12 weeks, 5-6 months, and >8 months. Diameter and flow ratios and bifurcation angles were determined in each bifurcation of the LCA trees.

**Results:** The blood volume and inlet flow rate of LCA trees increase and decrease during juvenile and adult growth, respectively. As vessel diameters decrease, the increased ratios of small to large daughter vessel diameters (*D*_*s*_/*D*_*l*_) result in more uniform flows and lower velocities. There are significant structure-functional changes of LCA trees in mice of >8 months compared with mice of < 8 months. As *D*_*s*_/*D*_*l*_ increases, the variation trend of bifurcation angle during juvenile growth is different from that during adult growth.

**Conclusions:** Although inlet flows are different in adult vs. juvenile mice, the adult still have uniform flow and low velocity. This is accomplished through a decrease in diameter. The design ensures ordered dispersion of red cells through asymmetric branching patterns into the capillaries.

## Introduction

The structure and function of coronary arterial trees undergo changes during normal growth and aging (Wei, 1992; LeBlanc and Hoying, 2016). For example, an increase of vessel density was found in the adult primarily owing to angiogenesis in which new daughter vessel segments grow (sprouting) or split (intussusception) from existing mother segments (Carmeliet and Jain, 2011; LeBlanc and Hoying, 2016). We have recently shown an age-independent exponent in the length-volume scaling law of an entire coronary arterial tree in juvenile and adult mice (Chen et al., 2015) because of fractal-like tree features (Huo and Kassab, 2016). In comparison with the unchanged “global” hierarchy of a vascular tree structure, the “local” branching patterns characterize the age-dependent anatomy of coronary arterial trees and affect flow patterns at junctions (Huo et al., 2009a; Huo Y. L. et al., 2012). The change of flow patterns can lead to low wall shear stress, high oscillatory shear index, high spacial gradient of wall shear stress, and so on (Huo et al., 2007a, 2008, 2009b). These hemodynamic parameters are related to stagnation, reversal and vortical flows (Asakura and Karino, 1990; Kleinstreuer et al., 2001; Huo et al., 2007a), which result in abnormal biological responses such as dysfunction of endothelial cells, monocyte deposition, elevated wall permeability to macromolecules, particle migration into the vessel wall, smooth muscle cell proliferation, microemboli formation, and so on (Malek et al., 1999; Chiu and Chien, 2011). The current studies of advancing age in the coronary vasculature are generally confined to large epicardial arteries (LeBlanc and Hoying, 2016). To our knowledge, there is, however, lack of studies to show the effects of normal growth and development on the “local” branching patterns in coronary resistance vasculature (vessel diameter <200 μm).

Coronary blood flows in arterioles and small arteries play a fundamental role for regulation of total vascular resistance under physiological and pathological conditions (Chilian et al., 1989; Chilian, 1991; Pries and Secomb, 2005; Reglin et al., 2017). The flows are affected by multiple factors, e.g., Fahraeus-Lindqvist effects, bifurcation laws, and so on (Pries et al., 1995; Gompper and Fedosov, 2016; Secomb, 2017). Based on the constructal law, Bejan and Lorente indicated that an efficient transport system requires more symmetric bifurcations to keep fractional flows as uniform as possible (Bejan and Lorente, 2010). According to the morphometric measurements, normal arteriolar bifurcations are more symmetric than ischemia-regenerated or tumor-induced branching patterns to ensure ordered dispersion of red cells though the capillary network (Baish and Jain, 2000; Arpino et al., 2017).

The objective of the study is to investigate the changes of bifurcation asymmetry in coronary arterial trees of mice during juvenile and adult growth. We hypothesize that the bifurcation changes of small coronary arteries (i.e., diameter ratio and bifurcation angle) result in more uniform flows and lower velocities as vessel diameters decrease in mice of different ages. The inlet flow rate and blood volume of coronary arterial trees are also assumed to have different variation trends between juvenile and adult growth. To test the hypothesis, we analyzed diameter ratios and bifurcation angles in each bifurcation of coronary arterial trees reconstructed from Micro-CT (μCT) images of mice at ages of 3, 6, 12 weeks, 5-6 months and >8 months. Pulsatile blood flows in each tree were computed from a Womersley-type model (Huo and Kassab, 2006), based on which the flow and velocity ratios were determined in each bifurcation. The significance and limitation of the morphometric and hemodynamic analysis were discussed relevant to the microcirculation.

## Materials and methods

### Morphometric data

We have reconstructed coronary arterial trees of ICR (Institute of Cancer Research) mice from μCT images. All animal experiments were performed in accordance with Chinese National and Hebei University ethical guidelines regarding the use of animals in research, consistent with the NIH guidelines (Guide for the care and use of laboratory animals) on the protection of animals used for scientific purposes. The experimental protocols were approved by the Animal Care and Use Committee of Hebei University, China.

**Figure 1:**
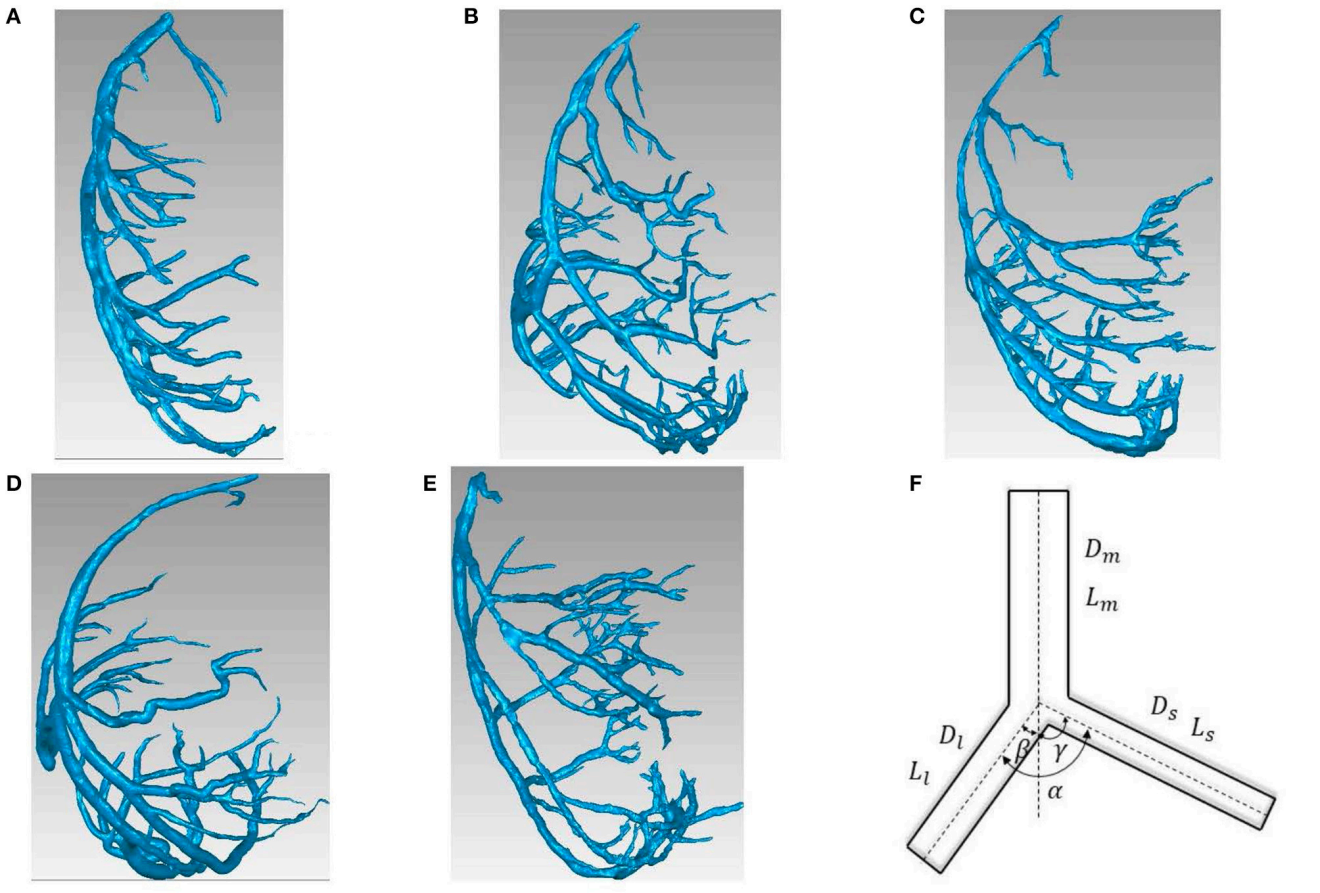
**(A-E)** LCA trees of mice at ages of 3 weeks **(A)**, 6 weeks **(B)**, 12 weeks **(C)**, 5-6 months **(D)** and >8 months **(E)** reconstructed from μCT images; **(F)** Schematic representation of a coronary bifurcation.

The reconstructed left coronary arterial (LCA) trees (including 9 LCA trees in 3 weeks group, 9 LCA trees in 6 weeks group, 7 LCA trees in 12 weeks group, 8 LCA trees in 5-6 months group and 9 LCA trees in > 8 months group) were used to analyze the changes in bifurcations of normal mice at different ages (from 3 weeks to >8 months), as shown in Figures 1A-E. Similar to a previous study (Chen et al., 2015), animals were anesthetized with pentobarbital sodium (60 mg/Kg) and heparinized with undiluted heparin (1 ml, 1,000 USPU/ml). After midline incision for laparotomy, animals were terminated by injecting an overdose of pentobarbital sodium through the inferior vena cava. The thoracic aorta was perfused with MICROFIL (Flow Tech, Carver, MA) at a constant pressure of 100 mmHg after the termination. The flow of cast solution was zero during the 90 min prior to hardening of cast at a constant pressure of 100 mmHg. The animal was stored in 10% formalin in the refrigerator for 24 h. The hearts were dissected and stored in 10% formalin in refrigerator until μCT scans. Morphometric data of LCA trees (including the diameter and rectangular coordinates of center points which were located in the center on the cross–sectional views of the contour of the 3D vessel) were extracted from μCT images using a gray-scale threshold method (with a low CT-threshold of 100) in the MIMICS software (Materialize, NV, Belgium).

A centerline was formed by a series of center points. Subsequently, the best fit diameter, *D*_*fit*_, was calculated as twice the average radius between the center point and the contour forming the 3D vessel. The blurring of small vessel edges was corrected to yield *D*_*correct*_ by fitting a Gaussian distribution function to the line profiles followed by computation of the input square wave. Since a vessel (a segment between two nodes of bifurcation) included 10-80 center points, the length and volume of a vessel were defined as: $L=\sum_{i=0}^{N-1}\sqrt{{\left(x_{i+1}-x_{i}\right)}^{2}+{\left(y_{i+1}-y_{i}\right)}^{2}+{\left(z_{i+1}-z_{i}\right)}^{2}}$ and $V=\sum_{i=0}^{N-1}\left(\frac{\pi D_{correct}^{2}}{4}.\sqrt{{\left(x_{i+1}-x_{i}\right)}^{2}+{\left(y_{i+1}-y_{i}\right)}^{2}+{\left(z_{i+1}-z_{i}\right)}^{2}}\right)$, where (*x, y, z*) refers to rectangular coordinates of center points from inlet (*i* = 0) to outlet (*i* = *N*) of the vessel. The cross-section area (CSA) of a vessel equaled to the intravascular volume divided by the length. A linear least-squares fit of all center points was used to determine the spatial direction of a vessel. The mother, large daughter, and small daughter diameters and lengths, (*D*_*m*_, *L*_*m*_), (*D*_*l*_, *L*_*l*_), and (*D*_*s*_, *L*_*s*_), as well as bifurcation angles were determined at all bifurcations of coronary arterial trees, as shown in Figure 1F. The LCA trees with vessel diameter ≥ 40 μm (twice the voxel size) were used to reduce the sampling error of the finite discrete grid. Unless otherwise stated, the terminal vessels of μCT-determined LCA trees have diameter ≥ 40 μm.

### Womersley-type model

Similar to a previous study (Huo and Kassab, 2006), a mathematical model is used to analyze pulsatile blood flow of coronary arteries in diastole in the absence of vessel tone. The governing equations for flow and pressure in a vessel (x = 0 and x = *L* refer to the inlet and outlet, respectively) can be written as:

(1) $$\begin{array}{l} Q\left(x,\omega \right)=a\cdot \text{cos}(\frac{\omega x}{c})\;+\;b\cdot \text{sin}\left(\frac{\omega x}{c}\right) \end{array}$$

(2) $$\begin{array}{l} P\left(x,\omega \right)=iZ_{1}[-a\cdot \text{sin}(\frac{\omega x}{c})\;+\;b\cdot \text{cos}(\frac{\omega x}{c})] \end{array}$$

where *a* and *b* are arbitrary constants of integration, ω the angular frequency, $c=\sqrt{1-F_{10}\left(ά\right)}\cdot c_{0}$ ($c_{0}=\sqrt{\frac{Eh}{\rho R}}$ and ά is the Womersley number) the wave velocity, $Y_{0}=\frac{A\left(n\right)}{\rho c_{0}}$ the characteristic admittance, $Z_{0}=\frac{1}{Y_{0}}$ the characteristic impedance, and $Z_{1}=Z_{0}/\sqrt{1-F_{10}\left(ά\right)}$. Moreover, we define the impedance and admittance as:

(3) $$\begin{array}{l} Z\left(x,\omega \right)=\frac{P\left(x,\omega \right)}{Q\left(x,\omega \right)}\text{ and }Y\left(x,\omega \right)=\frac{Q\left(x,\omega \right)}{P\left(x,\omega \right)} \end{array}$$

In a given vessel segment, at x = 0 and x = *L*, we have the respective inlet and outlet impedances:

(4) $$\begin{array}{l} Z\left(0,\omega \right)=\frac{iZ_{1}\cdot b}{a}\text{ and}\\ Z\left(L,\omega \right)=\frac{iZ_{1}\left[-a\cdot \sin\left(\frac{\omega L}{c}\right)+b\cdot \text{cos}\left(\frac{\omega L}{c}\right)\right]}{a\cdot \cos\left(\frac{\omega L}{c}\right)+b\cdot \text{sin}\left(\frac{\omega L}{c}\right)} \end{array}$$

From Equation (4), we obtain:

(5) $$\begin{array}{l} Z\left(0,\omega \right)=\frac{iZ_{1}\cdot \sin\left(\frac{\omega L}{c}\right)+Z\left(L,\omega \right)\cdot \text{cos}\left(\frac{\omega L}{c}\right)}{\text{cos}\left(\frac{\omega L}{c}\right)+iY_{1}\cdot Z\left(L,\omega \right)\cdot \sin\left(\frac{\omega L}{c}\right)} \end{array}$$

Equation (5) was used to calculate the impedance/admittance in a tree from inlet to the terminal vessels.

### Method of solution

The characteristic impedance, characteristic admittance and velocity (including the viscous effect) were first calculated for every vessel segment. We assume that mass is conserved and pressure is continuous at each bifurcation, which may be written as:

(6) $$\begin{array}{l} Q_{m}\left(\omega \right)=Q_{l}\left(\omega \right)+Q_{s}\left(\omega \right)\text{ and }P_{m}\left(\omega \right)=P_{l}\left(\omega \right)=P_{s}\left(\omega \right) \end{array}$$

From Equation (6), we obtain:

(7) $$\begin{array}{l} Y_{m}\left(L,\omega \right)=Y_{l}\left(0,\omega \right)+Y_{s}\left(0,\omega \right) \end{array}$$

Once the terminal impedance/admittance is computed, we proceed backwards to iteratively calculate the impedance/admittance in the entire coronary tree by using Equations (5) and (7) similar to a previous study (Huo and Kassab, 2006). The aortic pressure was obtained from a previous study (Huo et al., 2008) and discretized by a Fourier transformation to determine the constants *a* and *b* in Equations (1) and (2). The flow and pressure were then calculated by using Equations (1) and (2).

The blood flow density (ρ) in coronary arteries was assumed to be 1.06 g/cm^3^ (Chen et al., 2016; Fan et al., 2016; Yin et al., 2016). The variation of viscosity (μ) with vessel diameter and hematocrit was based on Pries' viscosity model (Pries et al., 1992). The coronary wall thickness was assumed to be one-tenth of the vessel diameter. The static Young's modulus was ~8.0 × 10^6^ (dynes/cm^2^) and the dynamic Young's modulus was also considered consistent with the previous study (Huo and Kassab, 2006). Symmetric arteriolar subtrees were pasted to all terminal vessels. A symmetric arteriolar subtree was constructed from the terminal vessel down to the first capillaries, based on two scaling relationships, $DR=2^{\frac{-1}{1.07+2}}$ and $LR=2^{\frac{-1}{3-0.42}}$ (DR and LR refer to the diameter and length ratio) (see Table 2 in Huo and Kassab, 2012a). The outlet impedances at the first capillaries were computed by the steady value; i.e., $\frac{128\mu_{capillary}L_{capillary}}{\pi D_{capillary}^{4}}$ (g · sec/cm^4^).

### Statistical analysis

The fraction of volumetric blood flow is mainly determined by diameter ratios ($\frac{D_{l}}{D_{m}}$, $\frac{D_{s}}{D_{m}}$, and $\frac{D_{s}}{D_{l}}$) while the change of flow velocities from mother to daughter vessels is characterized by the area expansion ratio ($AER=\frac{D_{l}^{2}+D_{s}^{2}}{D_{m}^{2}}$) (VanBavel and Spaan, 1992; Kaimovitz et al., 2008). Bifurcation angle in small arteries regulates the spacial heterogeneity of coronary blood flow albeit it is a critical risk factor for atherosclerotic plaques and stenting restenosis in large epicardial coronary arteries (Huo et al., 2012b; Huo Y. et al., 2012a). Hence, similar to previous studies (Huo et al., 2007b; Huo and Kassab, 2012b), diameter ratios ($\frac{D_{l}}{D_{m}}$, $\frac{D_{s}}{D_{m}}$, and $\frac{D_{s}}{D_{l}}$), area expansion ratios ($AER=\frac{D_{l}^{2}+D_{s}^{2}}{D_{m}^{2}}$), flow ratios ($\frac{Q_{l}}{Q_{m}}$, $\frac{Q_{s}}{Q_{m}}$, and $\frac{Q_{s}}{Q_{l}}$), and velocity ratios ($\frac{V_{l}}{V_{m}}$, $\frac{V_{s}}{V_{m}}$, and $\frac{V_{s}}{V_{l}}$) in a LCA tree were analyzed in eight mother diameter (i.e., *D*_*m*_) ranges as: Range 1 (<100 μm), Range 2 (100-120 μm), Range 3 (120-140 μm), Range 4 (140-160 μm), Range 5 (160-180 μm), Range 6 (180-200 μm), Range 7 (200-250 μm) and Range 8 (≥250 μm). Moreover, bifurcation angles in a LCA tree were summarized in four $\frac{D_{s}}{D_{l}}$ ranges as: Range 1 (<0.4), Range 2 (0.4-0.6), Range 3 (0.6-0.8) and Range 4 (≥0.8). The mean and standard deviation (mean±SD) were computed by averaging over all bifurcations in each group. Two Way Repeated Measures ANOVA (SigmaStat 3.5) was used to compare those morphometric and hemodynamic parameters in bifurcations between different ages and between different diameter ranges, where *p* < 0.05 represented a statistically significant difference.

## Results

**Figure 2:**
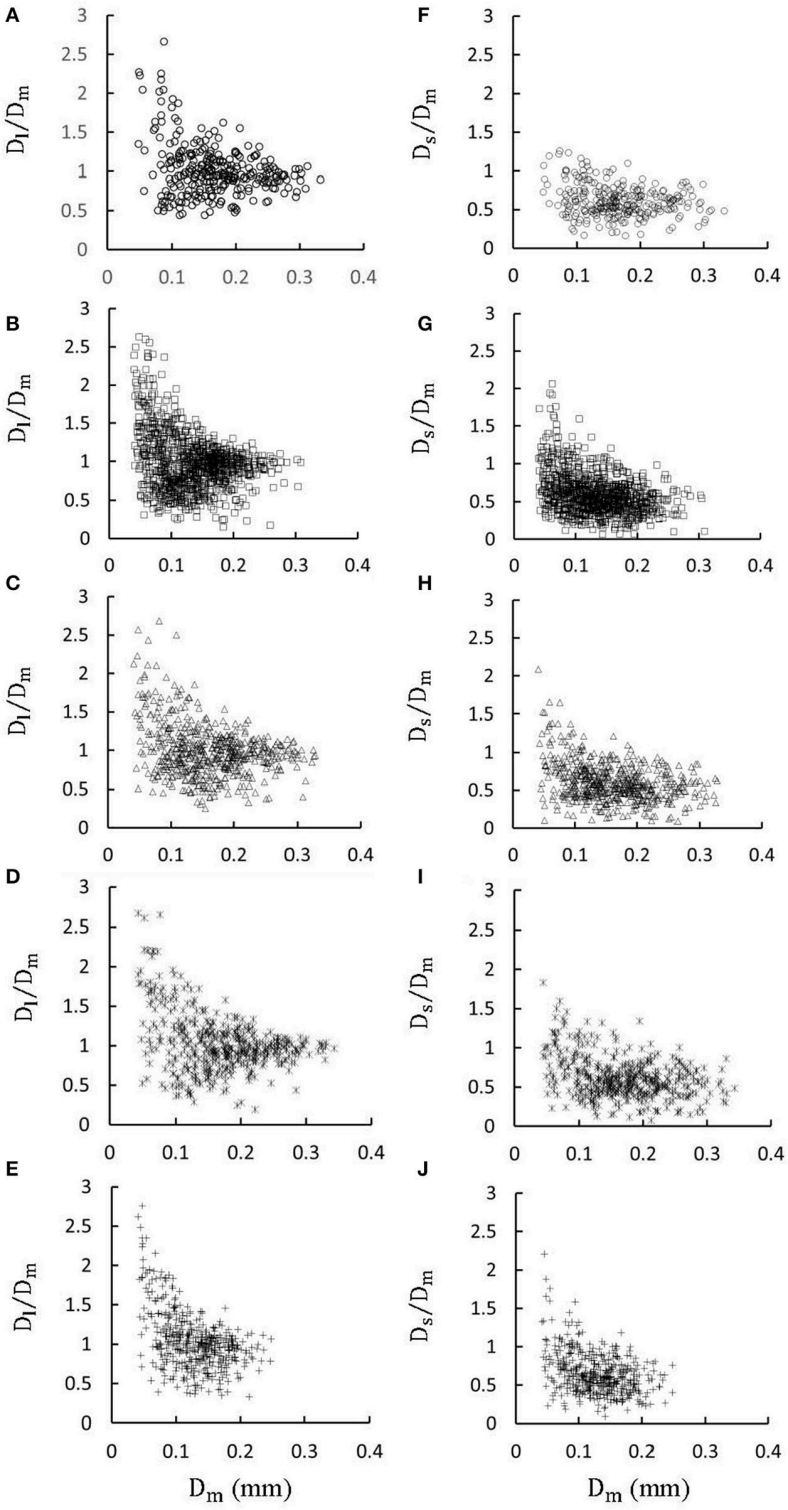
**(A–E)** Relationship between *D*_*l*_/*D*_*m*_ (diameter ratio of large daughter to mother vessels) and *D*_*m*_ (diameter of mother vessel) in all bifurcations of mice at ages of 3 weeks **(A)**, 6 weeks **(B)**, 12 weeks **(C)**, 5-6 months **(D)** and >8 months **(E)**; **(F-J)** Relationship between *D*_*s*_/*D*_*m*_ (diameter ratio of small daughter to mother vessels) and *D*_*m*_ in all bifurcations of mice at ages of 3 weeks **(F)**, 6 weeks **(G)**, 12 weeks **(H)**, 5-6 months **(I)**, and >8 months **(J)**. There is significant difference of *D*_*l*_/*D*_*m*_ and *D*_*s*_/*D*_*m*_ between mice of >8 months and 3 weeks, between mice of >8 months and 6 weeks, between mice of >8 months and 12 weeks, and between mice of >8 months and 5-6 months while there is no statistical difference between mice of other ages.

**Figure 3:**
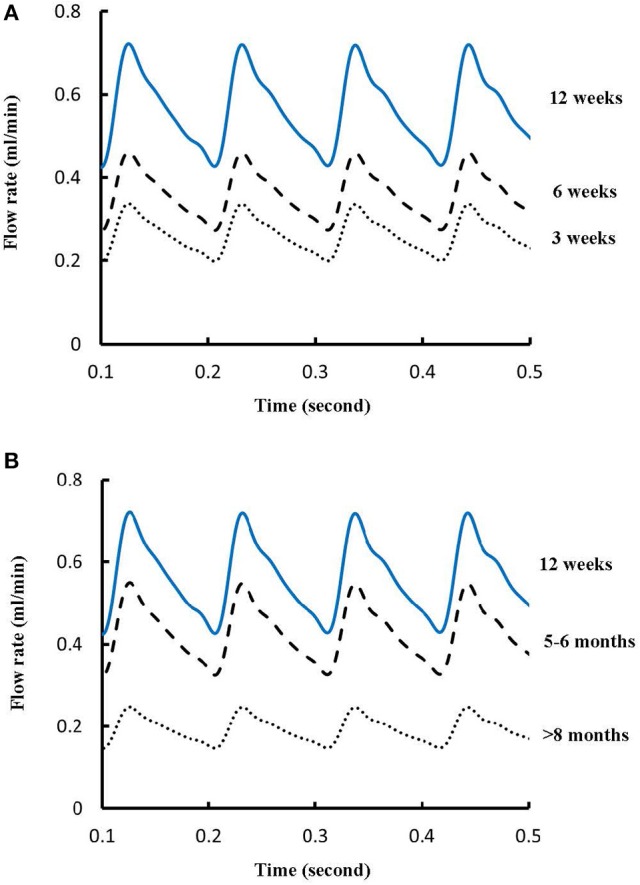
**(A)** Pulsatile blood flows at the inlet of LCA trees of mice during juvenile growth (from 3 to 12 weeks) and **(B)** Pulsatile blood flows at the inlet of LCA trees of mice during adult growth (≥12 weeks). The mean ± SD values of time-averaged flow rates over a cardiac cycle equal to 0.26 ± 0.17, 0.36 ± 0.23, 0.51 ± 0.18, 0.43 ± 0.22, and 0.21 ± 0.15 ml/min for mice at ages of 3, 6, 12 weeks, 5-6 months and >8 months, respectively.

Figures 1A-E show LCA trees of mice at ages of 3, 6, 12 weeks, 5-6 months, and >8 months reconstructed from μCT images. Accordingly, the LCA trees have blood volumes of 7.1 ± 4.1, 9.9 ± 3.7, 16.1 ± 4.7, 13.8 ± 4.0, and 5.5 ± 3.4 (× 10^−4^) cm^3^ (averaged over all LCA trees in each group) and the animals have body weights (BW) of 11.0 ± 0.8, 17.4 ± 1.5, 37.6 ± 3.9, 36.1 ± 4.1, and 38.1 ± 4.8 g (averaged over all animals in each group). Figures 2A-E show the changes of *D*_*l*_/*D*_*m*_ (diameter ratio of large daughter to mother vessels) as a function of *D*_*m*_ (diameter of mother vessel) in all bifurcations of mice at different age groups. Figures 2F-J show the corresponding changes of *D*_*s*_/*D*_*m*_ (diameter ratio of small daughter to mother vessels) with *D*_*m*_. A comparison in all bifurcations shows that mice of >8 months have significant difference of *D*_*l*_/*D*_*m*_ and *D*_*s*_/*D*_*m*_ from mice of <8 months (*p* < 0.05 between mice of >8 months and 3 weeks, between mice of >8 months and 6 weeks, between mice of >8 months and 12 weeks, and between mice of >8 months and 5-6 months) despite no statistical difference between mice of other ages. Figures 3A,B show pulsatile blood flows (waves averaged over all mice at the same age) at the inlet of LCA trees of mice during juvenile and adult growth, respectively. The time-averaged flow rate over a cardiac cycle has mean ± SD values of 0.26 ± 0.17, 0.36 ± 0.23, 0.51 ± 0.18, 0.43 ± 0.22, and 0.21 ± 0.15 ml/min for mice at ages of 3, 6, 12 weeks, 5-6 months and >8 months, which are proportional to the blood volumes of LCA trees. Similar to the changes of *D*_*l*_/*D*_*m*_ and *D*_*s*_/*D*_*m*_, a comparison in all bifurcations shows mice of >8 months have significant difference of *Q*_*l*_/*Q*_*m*_ and *Q*_*s*_/*Q*_*m*_ from mice of <8 months despite no statistical difference between mice of other ages. Moreover, there is significant difference of *V*_*l*_/*V*_*m*_ and *V*_*s*_/*V*_*m*_ between mice of 3 and 6 weeks, between mice of 3 and 12 weeks, and between mice of 3 weeks and 5-6 months as well as between mice of >8 months and 6 weeks, between mice of >8 months and 12 weeks, and between mice of >8 months and 5-6 months.

**Figure 4:**
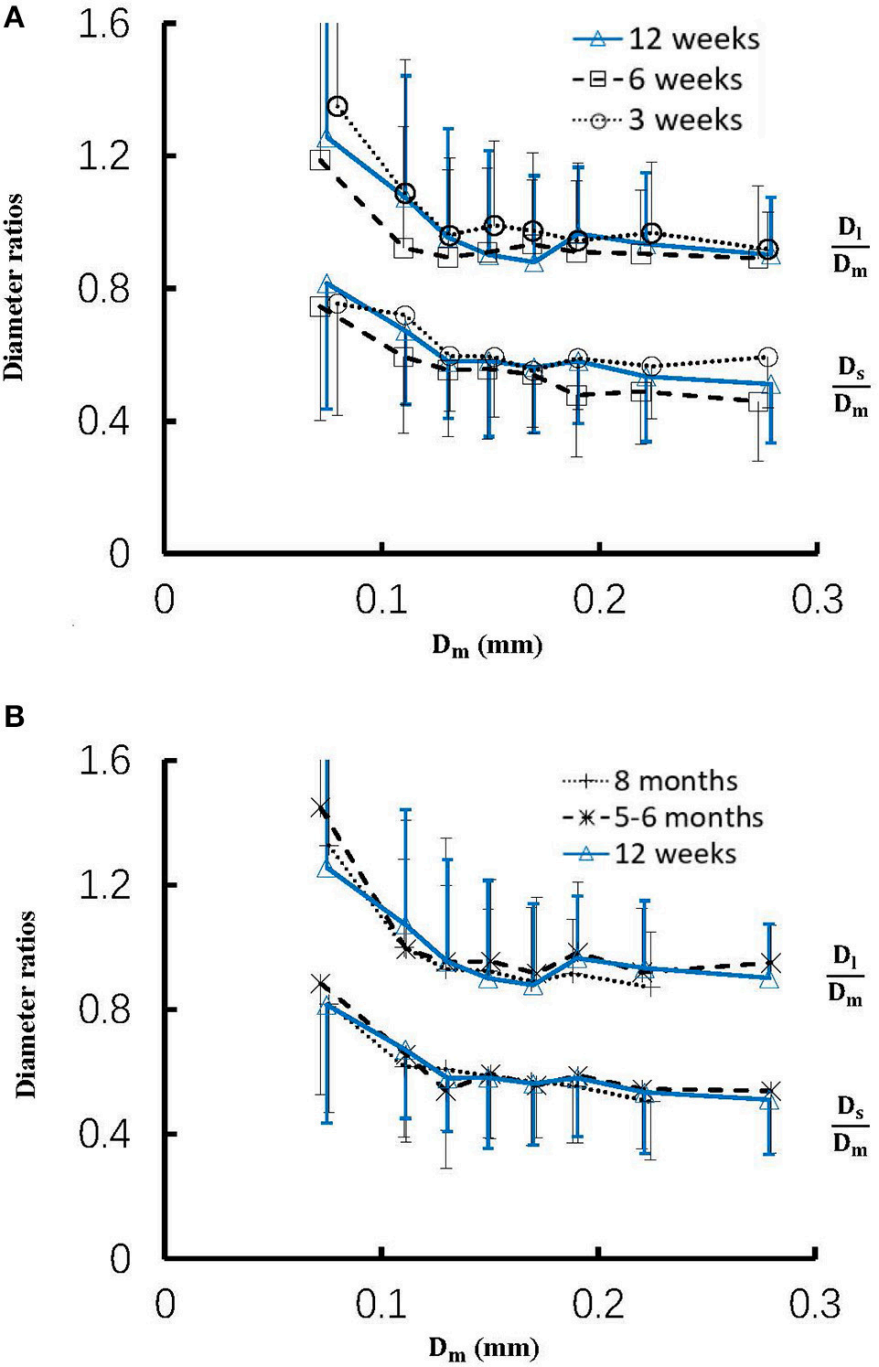
**(A)** Relationship between *D*_*l*_/*D*_*m*_, *D*_*s*_/*D*_*m*_, and *D*_*m*_ in 8 diameter ranges of coronary arterial trees of mice during juvenile growth (from 3 to 12 weeks) and **(B)** Relationship between *D*_*l*_/*D*_*m*_, *D*_*s*_/*D*_*m*_, and *D*_*m*_ in 8 diameter ranges of coronary arterial trees of mice during adult growth (≥12 weeks). Error bars refer to the SDs of those parameters in each diameter ranges. There is no statistical difference of *D*_*l*_/*D*_*m*_ and *D*_*s*_/*D*_*m*_ between mice of various ages in each single diameter range. For mice of all ages, there is significant difference of *D*_*l*_/*D*_*m*_ and *D*_*s*_/*D*_*m*_ between range 1 and other ranges and between range 2 and other ranges.

**Figure 5:**
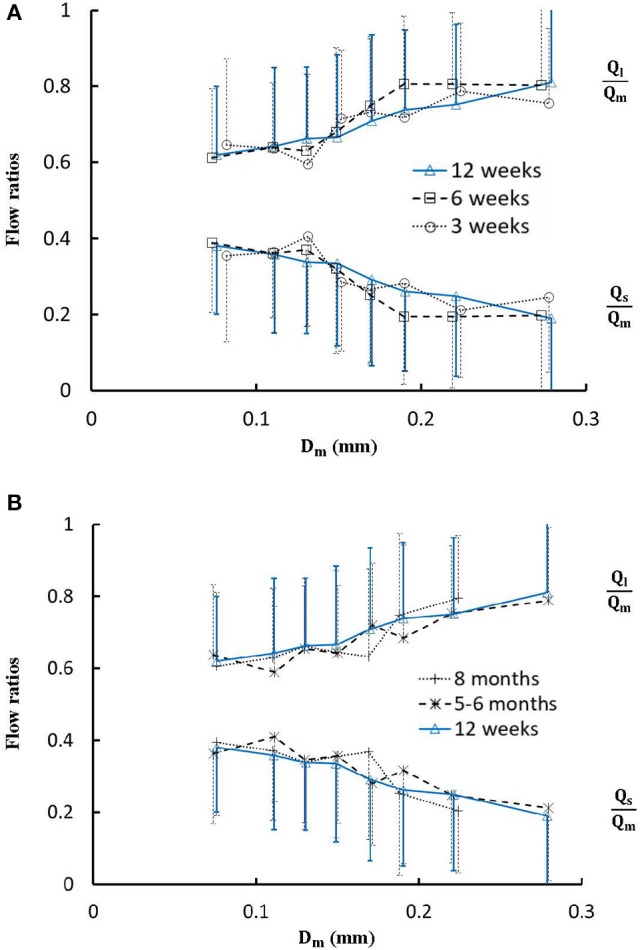
**(A)** Relationship between *Q*_*l*_/*Q*_*m*_, *Q*_*s*_/*Q*_*m*_, and *D*_*m*_ in 8 diameter ranges of coronary arterial trees of mice during juvenile growth (from 3 to 12 weeks) and **(B)** Relationship between *Q*_*l*_/*Q*_*m*_, *Q*_*s*_/*Q*_*m*_, and *D*_*m*_ in 8 diameter ranges of coronary arterial trees of mice during adult growth (≥12 weeks). Error bars refer to the SDs of those parameters in each diameter ranges. There is no statistical difference of *Q*_*l*_/*Q*_*m*_ and *Q*_*s*_/*Q*_*m*_ between mice of various ages in each single diameter range. For mice of all ages, there is no statistical difference between different ranges.

**Figure 6:**
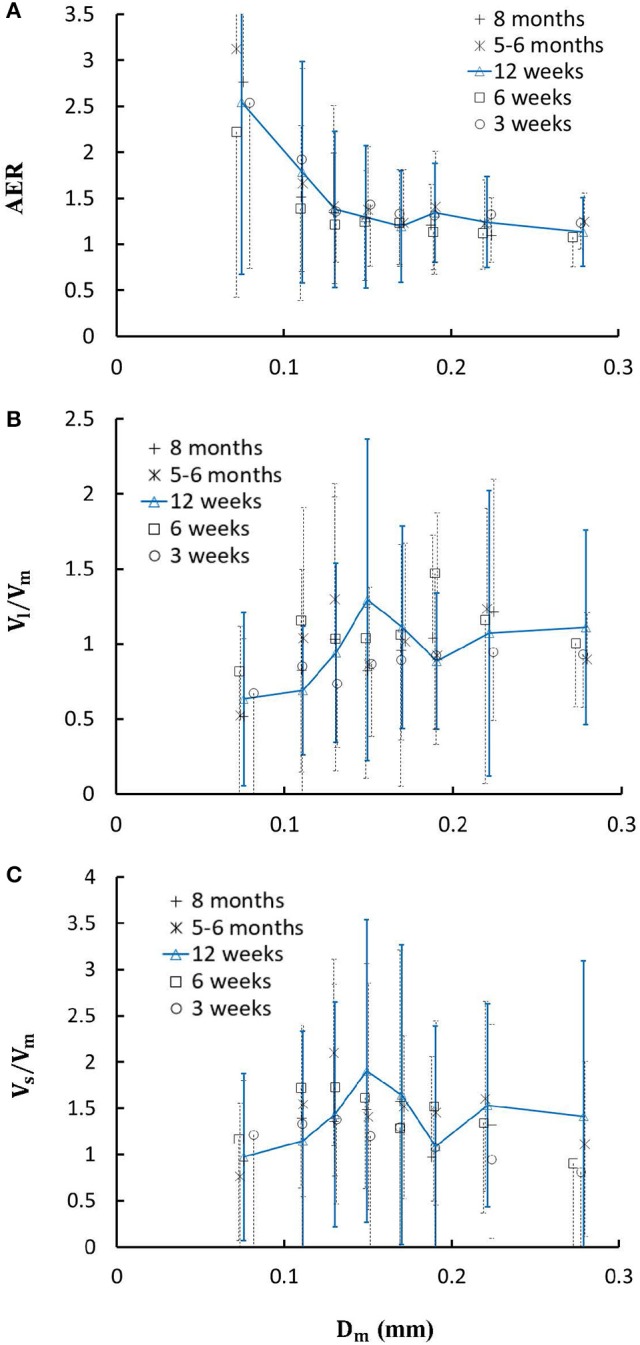
**(A)** Relationship between area expansion ratio ($AER=\frac{D_{l}^{2}+D_{s}^{2}}{D_{m}^{2}}$) and *D*_*m*_; **(B)** Relationship between *V*_*l*_/*V*_*m*_ and *D*_*m*_; and **(C)** Relationship between *V*_*s*_/*V*_*m*_ and *D*_*m*_ in 8 diameter ranges of coronary arterial trees of mice during juvenile and adult growth. Error bars refer to the SDs of those parameters in each diameter ranges. For mice of all ages, there is significant difference of AER, *V*_*l*_/*V*_*m*_ and *V*_*s*_/*V*_*m*_ between range 1 and other ranges and between range 2 and other ranges.

Figure 4 shows the changes of diameter ratios as a function of mother diameter during juvenile and adult growth while Figure 5 shows the changes of flow ratios. Figures 6A-C show the relationships between AER and *D*_*m*_, between *V*_*l*_/*V*_*m*_ and *D*_*m*_, and between *V*_*s*_/*V*_*m*_ and *D*_*m*_ in coronary arterial trees of mice during juvenile and adult growth. A comparison of those parameters in each single diameter range shows no statistical difference between mice of different ages. As mother vessel diameter increases in mice of 3, 6, 12 weeks, 5-6 months and >8 months, AER and diameter ratios (*D*_*l*_/*D*_*m*_ and *D*_*s*_/*D*_*m*_) decrease abruptly when *D*_*m*_ < 140 μm and remain relatively unchanged when *D*_*m*_≥140 μm (*p* < 0.05 between range 1 and other ranges and between range 2 and other ranges). Moreover, *D*_*s*_/*D*_*l*_ values when *D*_*m*_ < 140 μm are slightly higher than those when *D*_*m*_>200 μm. There are gradual increase and decrease of *Q*_*l*_/*Q*_*m*_ and *Q*_*s*_/*Q*_*m*_, respectively, with the increase of mother vessel diameter. There is an abrupt decrease of *V*_*l*_/*V*_*m*_, and *V*_*s*_/*V*_*m*_ as mother vessel diameter decreases from 140 to 40 μm (*p* < 0.05 between range 1 and other ranges and between range 2 and other ranges).

**Figure 7:**
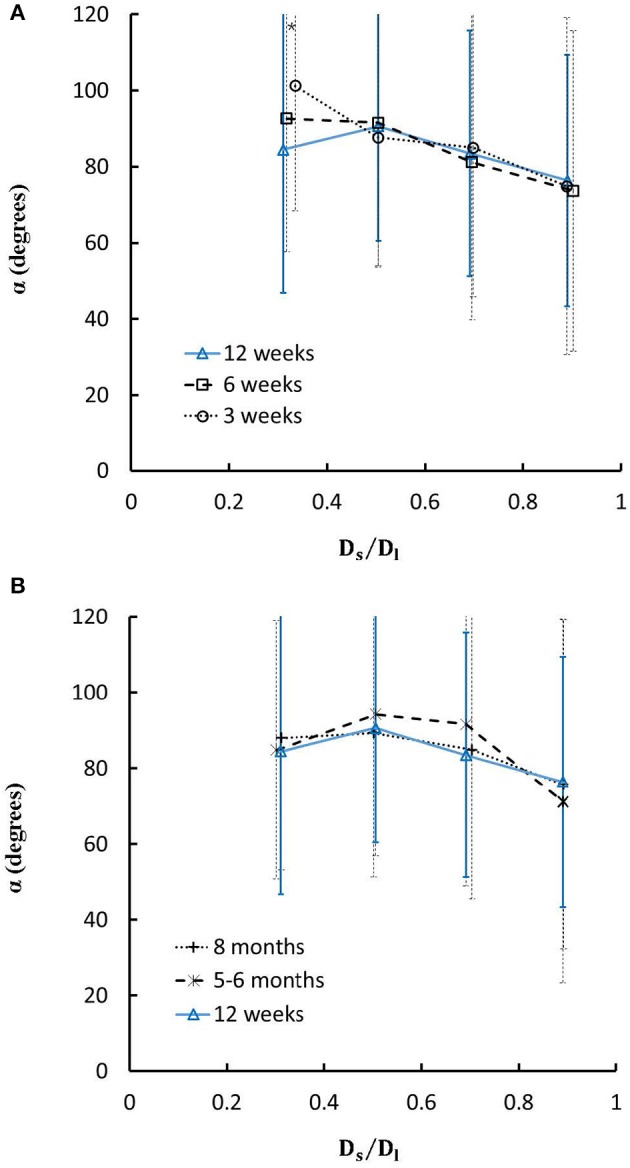
**(A)** Relationship between bifurcation angles and *D*_*s*_/*D*_*l*_ (diameter ratio of small to large daughter vessels) in mice during juvenile growth and **(B)** Relationship between bifurcation angles and *D*_*s*_/*D*_*l*_ in mice during adult growth. Error bars refer to the SDs of bifurcation angles in each *D*_*s*_/*D*_*l*_ ranges. Symbol “*” refers to the statistical difference in mice of 3 weeks vs. 6 weeks, 3 weeks vs. 12 weeks, 3 weeks vs. 5-6 months, and 3 weeks vs. >8 months.

On the other hand, Figures 7A,B show the relationships between the measured bifurcation angles (α_*measured*_) and *D*_*s*_/*D*_*l*_ during juvenile and adult growth, respectively. There is a monotonical decrease of α_*measured*_ with the increase of *D*_*s*_/*D*_*l*_ at ages of 3 and 6 weeks, but a parabolic curve with peak values (~90°) when *D*_*s*_/*D*_*l*_ = 0.5 at ages of 12 weeks, 5-6 months, and > 8 months (*p* < 0.05 for 3 weeks vs. 6 weeks, 3 weeks vs. 12 weeks, 3 weeks vs. 5-6 months, and 3 weeks vs. >8 months when *D*_*s*_/*D*_*l*_ < 0.4).

## Discussion

The present study used a Womersley-type mathematical model to compute pulsatile blood flows in LCA trees of mice. The computed flow rates at the inlet of LCA trees vary in the range of 0.2-0.5 ml/min in agreement with the Doppler measurements (Teng et al., 2016). The inlet flow rate and blood volume of LCA trees increase in mice from 3 to 12 weeks and decrease during adult growth. Myocardial flows were estimated to be 9.45, 8.28, 5.43, 4.76, and 2.21 ml/min/g, respectively, in mice of 3, 6, 12 weeks, 5-6 months and >8 months. Myocardial flows in mice of < 8 months are significantly higher than those in porcine (~2.25 mL/min/g in Huo et al., 2009a). On the other hand, the waveform is preserved at the inlet of LCA trees in diastole in the absence of vessel tone during juvenile and adult growth, as shown in Figure 3. Furthermore, Figure 5 showed the development of relatively uniform flows as vessel diameters decrease from the inlet to 40 μm in LCA trees of mice, which agrees with previous findings (Bejan and Lorente, 2010). This is mainly attributed to the design of branching patterns as shown in Figures 1, 4.

A key finding of the study is that diameter ratios (*D*_*l*_/*D*_*m*_ and *D*_*s*_/*D*_*m*_) and AER when 40 μm ≤ *D*_*m*_ < 140 μm in mice are significantly higher than those in porcine (Kaimovitz et al., 2008) despite their similarity at 140 μm ≤ *D*_*m*_ < 350 μm, which leads to an abrupt decrease of velocity ratios (*V*_*l*_/*V*_*m*_ and *V*_*s*_/*V*_*m*_) as vessel diameters decrease from 140 to 40 μm in Figure 6 (Kassab, 2005). Mice at the age of <8 months have significantly higher myocardial flows (>two-fold) than large animals. Owing to the proportional relation between myocardial flow and metabolic rate, a significant increase of diameter ratios and AER reduces the flow velocity in vessels of 40 μm ≤ *D*_*m*_ < 140 μm to satisfy the metabolism in the mouse heart compared with large animals in that microvascular blood flow per unit of time is to ensure the needed exchange of substances between tissue and blood compartments (Jacob et al., 2016). We also demonstrated a comparison of diameter and flow ratios to show the effects of normal growth and development on morphometry and hemodynamics of LCA trees of mice. The statistical analysis at all bifurcations showed significant difference of diameter and flow ratios between mice of >8 months and others as well as no statistical difference between mice of <8 months. Myocardial flows in mice of >8 months were also significantly lower than others. Diameter ranges 1-7 showed no statistical difference between mice of different ages while mice of >8 months have no arteries in diameter range 8 (≥250 μm). Hence, the structure-functional change of LCA trees in mice of >8 months is mainly attributed to the regression of blood vessels (blood volumes of 7.1 ± 4.1, 9.9 ± 3.7, 16.1 ± 4.7, and 13.8 ± 4.0 vs. 5.5 ± 3.4 (× 10^−4^) cm^3^).

On the other hand, we showed the linear relationship between bifurcation angles and *D*_*s*_/*D*_*l*_ in LCA trees of mice at ages of 3 and 6 weeks, but the parabolic curve with the peak bifurcation angles (~90°) at *D*_*s*_/*D*_*l*_ = 0.5 in mice at ages of 12 weeks, 5-6 months and > 8 months. The linear relationship may be caused by the progression of mouse heart during juvenile growth while the parabolic curve is associated with the mature and stable heart size during adult growth, which requires further investigations with considering how the spatial heterogeneity of myocardial flows is altered by the age-dependent bifurcation angles.

### Critique of the study

The present study carried out the pulsatile blood flow analysis in coronary arterial trees of mice during normal juvenile and adult growth, which brings in some complexities. For example, although coronary arterial trees with diameter > 40 μm were reconstructed from μCT images, symmetric arteriolar subtrees with diameters from 40 μm down to the first capillaries were generated from two scaling relationships. The simple Womersley-type model was derived for Newtonian fluids in straight pipes. Here, the non-Newtonian effect was partly captured in small arteries by using a diameter-dependent viscosity. Based on a more realistic model with considering non-Newtonian fluids, the hemodynamic analysis should be performed to accurately validate morphometric predictions when arteriolar trees with diameter <40 μm are available. Moreover, the following studies should relate vessel tone and metabolic signals to the 3D spatial bifurcation asymmetry for understanding the microcirculation and myocardial heterogeneity deeply.

## Conclusions

This study analyzed the morphometric and hemodynamic variation of microvascular bifurcations in LCA trees of normal mice at ages of 3, 6, 12 weeks, 5-6 months and >8 months. The inlet flow rate and blood volume of LCA trees increase during juvenile growth and decrease during adult growth while the flow waveform is preserved in diastole in the absence of vessel tone. The blood flow becomes more uniform as vessel diameters decrease from the inlet to 40 μm owning to the changes of diameter ratios (*D*_*l*_/*D*_*m*_ and *D*_*s*_/*D*_*m*_). The changes of diameter ratios and AER also lead to an abrupt decrease of velocity ratios with the decrease of vessel diameter from 140 to 40 μm. Mice of >8 months show structure-functional difference from others.

## Author contributions

The data analysis was done by YF, XW, TF, and XS. Micro-CT images were collected by LL, WZ, and MC. The manuscript was drafted and revised by YH, JLiu and JLi.

### Conflict of interest statement

The authors declare that the research was conducted in the absence of any commercial or financial relationships that could be construed as a potential conflict of interest.
